# Towards fast whole-body PET/MR: Investigation of PET image quality versus reduced PET acquisition times

**DOI:** 10.1371/journal.pone.0206573

**Published:** 2018-10-30

**Authors:** Maike E. Lindemann, Vanessa Stebner, Alexander Tschischka, Julian Kirchner, Lale Umutlu, Harald H. Quick

**Affiliations:** 1 High-Field and Hybrid MR Imaging, University Hospital Essen, University Duisburg-Essen, Essen, Germany; 2 Department of Nuclear Medicine, Medical Faculty, University Duisburg-Essen, Essen, Germany; 3 Department of Diagnostic and Interventional Radiology, University Hospital Düsseldorf, University of Düsseldorf, Düsseldorf, Germany; 4 Department of Diagnostic and Interventional Radiology and Neuroradiology, University Hospital Essen, University Duisburg-Essen, Essen, Germany; 5 Erwin L. Hahn Institute for Magnetic Resonance Imaging, University Duisburg-Essen, Essen, Germany; Lee Kong Chian School of Medicine, SINGAPORE

## Abstract

**Purpose:**

The trend towards faster acquisition protocols in whole-body positron emission tomography/magnetic resonance (PET/MR) arises the question of whether short PET data acquisition protocols in a whole-body multi-station context allow for reduced PET acquisition times while providing adequate PET image quality and accurate quantification parameters. The study goal is to investigate how reducing PET acquisition times affects PET image quality and quantification in whole-body PET/MR in patients with oncologic findings.

**Methods:**

Fifty-one patients with different oncologic findings underwent a clinical whole-body 18F-Fluorodeoxyglucose PET/MR examination. PET data was reconstructed with 4, 3, 2, and 1 min/bed time intervals for each patient to simulate the effect of reduced PET acquisition times. The 4-minute PET reconstructions served as reference standard. All whole-body PET data sets were analyzed regarding image quality, lesion detectability, PET quantification and standardized uptake values.

**Results:**

A total of 91 lesions were detected in the 4-minute PET reconstructions. The same number of congruent lesions was also noticed in the 3 and 2 minutes-per-bed (mpb) reconstructed images. A total of 2 lesions in 2 patients was not detected in the 1 minute PET data reconstructions due to poor image quality. Image noise in the blood pool increased from 22.2% (4 mpb) to 42.1% (1 mpb). Signal-to-noise ratio declined with shorter timeframes from 13.1 (4 mpb) to 9.3 (1 mpb). SUV_mean_ and SUV_max_ showed no significant changes between 4 and 1 mpb reconstructed timeframes.

**Conclusions:**

Reconstruction of PET data with different time intervals has shown that 2 minutes acquisition time per bed position instead of 4 minutes is sufficient to provide accurate lesion detection and adequate image quality in a clinical setting, despite the trends to lower image quality with shorter PET acquisition times. This provides latitude for potential reduction of PET acquisition times in fast PET/MR whole-body examinations.

## Introduction

The successful integration of positron emission tomography (PET) and magnetic resonance (MR) imaging into one whole-body PET/MR system combines the excellent soft-tissue contrast and several functional imaging parameters provided by MR with the high sensitivity and quantification of radiotracer metabolism supplied by PET [1] [2] [3]. From the clinical introduction of the new hybrid method [4] [5] [6], PET/MR imaging has shown great potential in various applications over the recent years.

An inherent advantage of PET/MR over PET/CT is that MR provides a wide range of soft tissue contrasts and potentially adds diagnostic information complementary to the PET data. However, in most PET/MR imaging protocols this comes at the at the cost of considerably increased PET/MR acquisition times when compared to PET/CT. This is because MR requires the acquisition of multiple different imaging sequences (e.g. T1, T2, diffusion weighted imaging) to generate a choice of soft tissue contrasts per bed position. The repeated acquisition of multiple sequences adds to the PET/MR acquisition times per bed position [3] [4] [5] [6]. In conventional PET/CT imaging, the data acquisition approximately lasts 2 minutes per bed position with PET being the time limiting factor. Standard clinical whole-body MR protocols today may last between 5–10 minutes per bed position or even longer depending on the clinical indication. A clinical routine whole-body PET/MR protocol including 4–5 bed positions with a duration of 5–10 minutes per bed position mainly consists of five MR sequences (T1-weighted Dixon-VIBE, diffusion weighted EPI, T2-weighted TIRM, T2-weighted HASTE and T1-weighted VIBE post contrast). This prolonged whole-body MR protocol ensures different soft tissue for diagnostic assessment in a variety of oncologic indications. Thus, in such rather lengthy protocols, MR is currently the time limiting factor in PET/MR imaging.

Long acquisition times for whole-body PET/MR have been identified as a main limitation of PET/MR vs. PET/CT, reducing patient comfort and patient throughput [7]. Since the clinical introduction of PET/MR, investigators have worked on the optimization of the whole-body PET/MR imaging workflow and on shortening of the overall exam duration [8] [9] [10]. Recent efforts have now demonstrated that defined hybrid imaging protocols allow for further reduced MR imaging protocols [11] [12] [13] [14] [15] [16]. In these studies, sequences and contrast weightings providing only redundant diagnostic information have been eliminated from the list of MR protocols while maintaining diagnostic PET/MR information in specific clinical settings. Thus, PET/MR imaging workflow was streamlined and a fast, but still diagnostic imaging protocol was realized in these studies [11] [12] [13]. Updated whole-body ultra-fast MR protocols in clinical routine have recently been shortened to 3 to 5 minutes per bed position and might fall within the scope of PET being the time limiting factor in PET/MR. In these ultra-fast MR protocols only three MR sequences are used for reading: a T1-weighted Dixon-VIBE for attenuation correction, and a T2-weighted HASTE and T1-weighted VIBE post contrast for high-resolution diagnostic MR imaging.

Ultimately, a whole-body PET/MR exam could be as fast as the acquisition of the MR-based attenuation correction (AC) lasts without adding further diagnostic MR sequences to the protocol. This has been first demonstrated in an initial study by Eiber et al. [17], where the MR-based AC images (Dixon sequence) have also been used as anatomic correlate to the simultaneous PET images. Conclusion of this early study was that the fast protocol using MR-based AC only provides comparable results to a fast low-dose PET/CT exam [17]. Such a whole-body PET/MR protocol theoretically could be as fast as 5x 19 seconds, which is the duration for a current standard five-bed, low spatial resolution MR-based Dixon AC protocol [3] [18].

New technical developments such as continuously moving table MRI [19] that provide seamless whole-body PET/MR at different table speeds, furthermore, allow for more flexibility in whole-body PET/MR data acquisition and may allow for fast whole-body PET/MR imaging protocols when combined with a selection of fast MR imaging sequences [19].

In this context and in preparation of future fast whole-body PET/MR imaging protocols, the question arises whether short PET data acquisition protocols allow for reduced acquisition time while providing adequate PET image quality and accurate quantification parameters. The purpose of this retrospective study, thus, is a systematical investigation of the PET image quality and quantification parameters of clinical oncologic whole-body PET/MR protocols using 18F-Fluorodeoxyglucose (FDG) as radiotracer regarding the impact of reduced PET acquisition times. List-mode format PET data of a larger cohort of 51 patients with oncologic findings who underwent whole-body PET/MR exams was retrospectively reconstructed into different time frames to systematically simulate reduced PET acquisition times. The PET data were then analyzed regarding image quality and quantification parameters. Another associated implication of this study is to investigate the potential of reducing the injected tracer dose while keeping PET acquisition times constant.

## Material and methods

### Patient population

In this retrospective study, 51 patients with different oncologic findings that underwent a whole-body PET/MR examination were included. The patient population consists of 27 female and 24 male patients (mean age 45.5 ± 20.3 years; mean BMI 25.5 ± 5.6 kg/m^2^). All patients underwent a clinical ^18^F-FDG PET/MR for staging/restaging of malignant diseases (17 lymphoma, 10 gynecological carcinoma, 7 cancer of unknown primary, 6 breast cancer, 5 rectal carcinoma and 8 others). Of these, 22 patients were referred to a PET/MR examination only. The other 29 patients first underwent a clinically indicated PET/CT with tracer injection and subsequently an additional PET/MR examination without additional tracer injection. This resulted in a broad range of post injection times at the start of the PET/MR examination. The study was conducted in conformance with the Declaration of Helsinki and approved by the Ethics Commission of the Medical Faculty of the University Duisburg-Essen (study number 11–4822-BO), and all patients provided written informed consent before examination. Detailed patient information are listed in Table 1.

**Table 1:** Detailed patient information.

# Patient	Sex	Age [years]	Height [cm]	Weight [kg]	BMI [kg/m^2^]	Tracer	Injection dosage [MBq]	Start of measurement [min p.i.]	Type of malignancy
1	M	22	180	70	21,6	^18^F-FDG	148	60	bone cancer
2	F	50	168	105	37,2	^18^F-FDG	212	41	uterus carcinoma
3	F	33	168	73	25,9	^18^F-FDG	140	86	vulva carcinoma
4	M	29	192	135	36,6	^18^F-FDG	174	72	Morbus Hodgin
5	M	36	172	70	23,7	^18^F-FDG	210	84	lymphoma
6	F	17	170	87	30,1	^18^F-FDG	252	57	Hodgin lymphoma
7	M	22	181	70	21,4	^18^F-FDG	280	65	Morbus Hodgin
8	M	15	187	66	18,9	^18^F-FDG	335	72	Morbus Hodgin
9	F	41	160	63	24,6	^18^F-FDG	131	60	cervix carcinoma
10	M	14	157	40	16,2	^18^F-FDG	216	63	Morbus Hodgin
11	F	59	150	75	33,3	^18^F-FDG	116	98	mamma carcinoma, ovarian cancer
12	F	61	164	58	21,6	^18^F-FDG	132	50	cervix carcinoma
13	M	13	158	43	17,2	^18^F-FDG	116	79	Morbus Hodgin
14	F	69	163	78	29,4	^18^F-FDG	138	53	uterus carcinoma
15	M	75	172	80	27,0	^18^F-FDG	203	85	colon cancer
16	M	11	137	24	12,8	^18^F-FDG	125	65	b-symptomatology
17	F	80	168	80	28,3	^18^F-FDG	234	63	adenom carcinoma, lymphoma
18	F	28	160	64	25,0	^18^F-FDG	238	54	cervix carcinoma
19	M	60	178	77	24,3	^18^F-FDG	218	69	colon cancer
20	M	14	150	44	19,6	^18^F-FDG	112	50	PTLD
21	F	33	173	120	40,1	^18^F-FDG	143	51	cervix carcinoma
22	F	16	178	74	23,4	^18^F-FDG	165	54	Morbus Hodgin
23	M	46	180	86	26,5	^18^F-FDG	269	203	lymphoma
24	F	74	156	78	32,1	^18^F-FDG	245	148	mamma carcinoma
25	M	22	180	108	33,3	^18^F-FDG	344	112	Morbus Hodgin
26	M	71	168	65	23,0	^18^F-FDG	262	139	Morbus Hodgin
27	F	40	165	78	28,7	^18^F-FDG	223	160	cancer of unknown primary
28	F	37	158	58	23,2	^18^F-FDG	208	157	lymphoma
29	F	28	173	60	20,0	^18^F-FDG	290	149	cervix carcinoma
30	F	34	170	74	25,6	^18^F-FDG	230	142	Morbus Hodgin
31	F	80	168	96	34,0	^18^F-FDG	271	117	lymphoma
32	F	59	165	75	27,5	^18^F-FDG	211	122	adrenal carcinoma
33	F	44	158	55	22,0	^18^F-FDG	195	114	mamma carcinoma
34	F	35	167	98	35,1	^18^F-FDG	176	126	mamma carcinoma
35	M	58	180	78	24,1	^18^F-FDG	248	150	cancer of unknown primary
36	M	52	180	67	20,7	^18^F-FDG	267	107	lymphoma
37	M	60	184	63	18,6	^18^F-FDG	350	117	cancer of unknown primary
38	F	70	165	57	20,9	^18^F-FDG	212	115	Burkitt lymphoma
39	M	51	190	75	20,8	^18^F-FDG	280	112	cancer of unknown primary
40	F	77	150	50	22,2	^18^F-FDG	187	119	lymphoma
41	F	63	161	59	22,8	^18^F-FDG	208	163	mamma carcinoma
42	M	65	173	76	25,4	^18^F-FDG	230	155	cancer of unknown primary
43	M	63	179	73	22,8	^18^F-FDG	245	116	bronchial carcinoma
44	F	58	168	67	23,7	^18^F-FDG	223	172	adenom carcinoma
45	M	18	186	100	28,9	^18^F-FDG	286	191	colon cancer
46	F	55	168	69	24,4	^18^F-FDG	220	132	mamma carcinoma
47	M	42	176	94	30,3	^18^F-FDG	286	133	Hodgin lymphoma
48	F	46	168	81	28,7	^18^F-FDG	215	160	ovarian cancer
49	M	51	180	85	26,2	^18^F-FDG	304	137	cancer of unknown primary
50	M	63	163	63	23,7	^18^F-FDG	209	120	lymphoma
51	F	63	153	65	27,8	^18^F-FDG	225	289	cancer of unknown primary
mean ± SD		45.5 ± 20.3			25.5 ± 5.6		218.8 ± 59.1	110 ± 49	

Statistically relevant data of the patient population including e.g. the body mass index (BMI) and injected activity of radiotracer 18F-Fluorodeoxyglucose (FDG). PTLD as type of malignancy means post-transplant lymphoproliferative disease. Patients #1–#22 had a PET/MR examination only. The other patients first underwent a PET/CT and subsequently a PET/MR exam.

### Image acquisition and reconstruction

All PET/MR examinations were performed on an integrated 3 Tesla whole-body PET/MR system (Biograph mMR, Siemens Healthcare GmbH, Erlangen, Germany). The PET/MR only exams started 65 ± 14 min (mean ± SD) after injection of radiotracer 18F-FDG. The PET/MR measurements subsequently to a PET/CT started 144 ± 36 min. The patients were administered an average radiotracer dosage of 218.8 ± 59.1 MBq (mean ± SD) (range 112–350 MBq).

PET/MRI examinations were performed in caudocranial direction; on average 4–5 bed positions per patient were acquired. For each bed position in each patient, PET and MR data was acquired simultaneously for 4 minutes. The PET data was sampled in list-mode format and was then retrospectively reconstructed to 4, 3, 2, and 1-minute time intervals to simulate decreasing PET acquisition times. The reconstructed subsets were generated using the identical start time. Decay-, scatter- and random correction were executed identically for all reconstructed subsets. The 4-minute PET data served as reference standard in this study. All PET data reconstruction was performed with e7 tools (Siemens Molecular Imaging, Knoxville, USA) using OP-OSEM algorithm with 3 iterations, 21 subsets and 4 mm Gaussian filter. The resulting PET images have matrix dimensions of 344x344x127 with a reconstructed image resolution of 2.09x2.09x2.03 mm³. In the retrospective data reconstructions, rigid hardware components of the PET/MR system were automatically attenuation corrected with e7 tools by using CT-based templates of the patient table, the radiofrequency (RF) spine array coil and the RF head-neck coil [20], as it is also the case for routine exams using the PET/MR system. Attenuation correction of the patient’s soft tissue was performed by using standard MR-based AC-map of the patient. Therefore, a two-point (fat, water) Dixon-VIBE sequence generates a four-compartment model of the patient’s tissues by segmenting into air, lung, fat and soft tissue for attenuation correction [18].

MRI data was acquired simultaneous to PET data. A standardized clinical whole-body PET/MR protocol includes 4–5 bed positions with a duration of 8–12 minutes per bed position mainly consists of five MR sequences. The used MR sequences and parameters per MR protocol may differ according to the individual indications of the patients. The MR protocol was implemented using following MR sequences (Table 2).

**Table 2:** MR sequence parameters.

sequence name	orientation	slice thickness [mm]	repetition time/echo time [ms]	flip angle [°]	field-of-view [mm]	matrix size	acquisition time [min:sec]
T1w Dixon-VIBE	coronal	3,12	3,6/1,23 and 2,46	10	500	192x79	0:19
DWI EPI	axial	5	9900/82	90	420	160x90	2:48
T2w TIRM	coronal	5	3190/55	60	450	384x216	2:57
T2w HASTE	axial	5	1500/117	160	450	320x211	1:06
T1w VIBE post contrast	axial	3,5	4,08/1,51	9	400	512x230	0:18

MR sequence parameters according to a whole-body PET/MR protocol using a T1-weighted (w) Dixon volume-interpolated breath-hold examination (VIBE), diffusion-weighted imaging (DWI) echo-planar imaging (EPI), T2w Turbo-Inversion Recovery-Magnitude (TIRM), T2w half Fourier acquisition single-shot turbo spin echo (HASTE) and T1w VIBE post contrast.

### Image analysis

All PET/MR data sets with different reconstructed time intervals for each patient (4, 3, 2, 1 min) were analyzed regarding image quality and lesion detectability, applying a 4-point scale. The 4-point image quality score (IQS), was defined as 0 = non-diagnostic, 1 = poor, 2 = moderate, and 3 = good. Additionally, the following quantitative PET imaging parameters were assessed: the standardized uptake values (SUV) SUV_mean_, SUV_max_ in all detectable lesions, in liver and in the mediastinum as well as signal-to-noise (SNR) and contrast-to-noise (CNR). Image reading was performed by two experienced readers, a radiologist and a nuclear medicine specialist. Reading was performed in consensus.

The PET images were analyzed starting with the 1 min/bed timeframe to 4 min/bed to avoid bias in lesion detectability. For quantitative evaluation SUV_mean_, SUV_max_ and SUV_SD_ were measured with OsiriX (Pixmeo SARL, Bernex, Switzerland) in patient’s background, liver and each lesion. Therefore, a region-of-interest (ROI) with a fixed diameter of 1.5 cm was generated in the descending aorta to measure the mediastinal blood pool as background. A ROI with a diameter of 1.5 cm was placed in the liver, which was chosen as an organ with homogeneous FDG uptake, but without any findings in each patient, serving as reference standard. Two additional ROIs (diameter 1.5 cm) were placed in the gluteus maximus and the spleen. Image noise in the liver, the gluteus maximus, the spleen and the blood pool were calculated. To quantify lesion detectability, SUVs were obtained in all recorded lesions in each patient and for each reconstruction. Volume-of-interest (VOI) around lesions were delineated using fixed threshold set to 40% of SUV_max_ in the lesion and SUVs were measured. All ROIs and VOIs were copied in identical planes and positions in each reconstructed time interval for each patient. SNR (1), CNR (2) and image noise for each time interval were calculated as described in detail by Yan et al. [21]:

$$SNR=\frac{{SUV}_{mean}lesion}{{SUV}_{SD}lesion}$$ (1)

$$CNR=\frac{{SUV}_{mean}lesion-{SUV}_{mean}background}{{SUV}_{SD}background}$$ (2)

Correlations and significance for image quality parameters (SNR, CNR, image noise) and potential determinants (acquisition time, dose, post injection time, BMI) were calculated. P-values < 0.05 were considered to be statistically significant.

## Results

### Image quality

**Fig 1:**
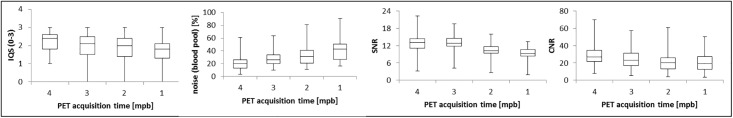
Boxplots of image quality parameter against PET acquisition time. Boxplots of the image quality score (IQS), which is defined as nondiagnostic = 0, poor = 1, moderate = 2 and good = 3; the image noise (blood pool), the signal-to-noise ratio (SNR) and contrast-to-noise ratio (CNR) against PET acquisition time in minutes per bed (mpb). Note that the image quality slightly but progressively decreases in each boxplot towards lower PET acquisition times. Also the SNR and CNR decrease with lower acquisition times, while the image noise increases.

The image quality analysis of different PET acquisition times is presented in Table 3. The image quality score decreases from 2.4 at 4 minutes per bed (mpb) over 2.1 (3 mpb) and 2.0 (2 mpb) to 1.8 at 1 mpb. Image noise in the blood pool increases from 22.2 ± 10.3% in the 4 min timeframe over 26.0 ± 12.2% (3 mpb) and 33.6 ± 10.6% (2 mpb) to 42.1 ± 14.9% in the 1 min timeframe. Also the calculated image noise in the spleen, the liver and the gluteus maximus increase with shorter acquisition times. The SNR also decreases with shorter time intervals from 13.1 (4 mpb) over 12.9 (3 mpb) and 10.4 (2 mpb) to 9.3 (1 mpb). The CNR decreases from 26.6 at 4 mpb over 22.9 (3 mpb) and 20.8 (2 mpb) to 20.5 at 1 mpb (Table 3). The image quality parameters SNR, CNR and image noise show no significant changes between 3 and 4 mpb acquisition time (p > 0.05), but significant changes in image quality with shorter PET acquisition times are observable (p < 0.05). SUV_mean_ shows no significant changes between 4 and 1 mpb (p > 0.05), but SUV_max_ shows significant changes between 4 and 1 mpb (p < 0.05) (Table 3). In Fig 1 the image quality scores IQS, image noise, SNR and CNR are shown in boxplots over the PET acquisition time. Note the decreasing trend in IQS, SNR and CNR from 4 to 1 mpb, while the image noise increases with shorter image duration.

**Table 3:** Quantitative and qualitative image analysis for different PET acquisition times.

PET acquisition time [mpb]	image quality score (grading 0–3)	image noise (blood pool) [%]	image noise (liver) [%]	image noise (spleen) [%]	image noise (gluteus maximus) [%]	SNR	CNR	SUVmean	SUV_max_
4	2,4 ± 1,1	22,2 ± 10,3 (3,2–60,0)	18,2 ± 6,8 (5,9–40,0)	16,4 ± 7,8 (6,1–51,3)	19,3 ± 10,1 (7,8–45,1)	13,1 ± 2,7 (3,2–22,1)	26,6 ± 8,1 (7,7–69,3)	7,2 ± 2,4 (1,8–27,1)	9,4 ± 6,2 (1,9–36,5)
3	2,1 ± 1,2	26,0 ± 12,2 (10,0–71,4)	19,3 ± 7,1 (7,1–40,0)	19,0 ± 6,9 (5,7–47,7)	23,7 ± 9,0 (10,1–51,0)	12,9 ± 2,1 (4,2–19,6)	22,9 ± 11,7 (5,5–57,1)	6,9 ± 1,7 (1,7–22,2)	9,4 ± 6,4 (1,9–36,5)
2	2,0 ± 1,2	33,6 ± 10,6 (11,1–83,3)	25,0 ± 9,9 (10,5–60,0)	22,6 ± 10,4 (12,5–50,8)	32,6 ± 11,5 (12,0–61,3)	10,4 ± 1,6 (2,7–16,0)	20,8 ± 9,2 (4,1–61,0)	7,0 ± 2,8 (1,1–17,4)	9,7 ± 6,4 (1,9–37,8)
1	1,8 ± 1,3	42,1 ± 14,9 (16,7–90,9)	32,9 ± 12,0 (13,3–60,0)	30,5 ± 13,1 (12,5–60,0)	49,5 ± 16,7 (18,3–70,4)	9,3 ± 1,9 (1,9–13,3)	20,5 ± 9,9 (3,4–51,6)	6,8 ± 2,2 (1,1–20,1)	10,2 ± 6,4 (2,0–37,0)

Lesion characterization and image quality analysis for different PET list-mode data reconstruction intervals. Values are presented in mean ± SD (range).

### Quantitative image parameters

A total of 91 lesions were detected in the 4 minute PET data sets serving as reference standard. Thereof, 20 lesions were located in head/neck area, 39 in the thorax region, 8 in the abdomen and 24 lesions in the pelvis or upper thighs. An identical number of congruent lesions were also noticed in the 3 and 2 mpb reconstructed PET images. In the 1-minute PET data reconstructions, overall 2 lesions in 2 patients could not be detected due to poor image quality. Patient #33 has a lymph node lesion in the left mammaria flow area, which could not be detected in the 1 minute timeframe. In this case, the patient’s BMI was 32 kg/m^2^, the applied radiotracer dosage was 3.1 MBq/kg and the post injection time was 148 minutes. The CNR is with 10.3 at 4 mpb significant lower than the average CNR (26.6). Patient #24 has a lymph node lesion left cervical, which also could not be detected in the 1 minute timeframe. In this second case, the patient’s BMI was 22 kg/m^2^, the applied radiotracer dosage was 3.5 MBq/kg and the post injection time was 114 minutes. The image noise here was with 58% in 1 mpb reconstruction significant higher than the average image noise (42%).

**Fig 2:**
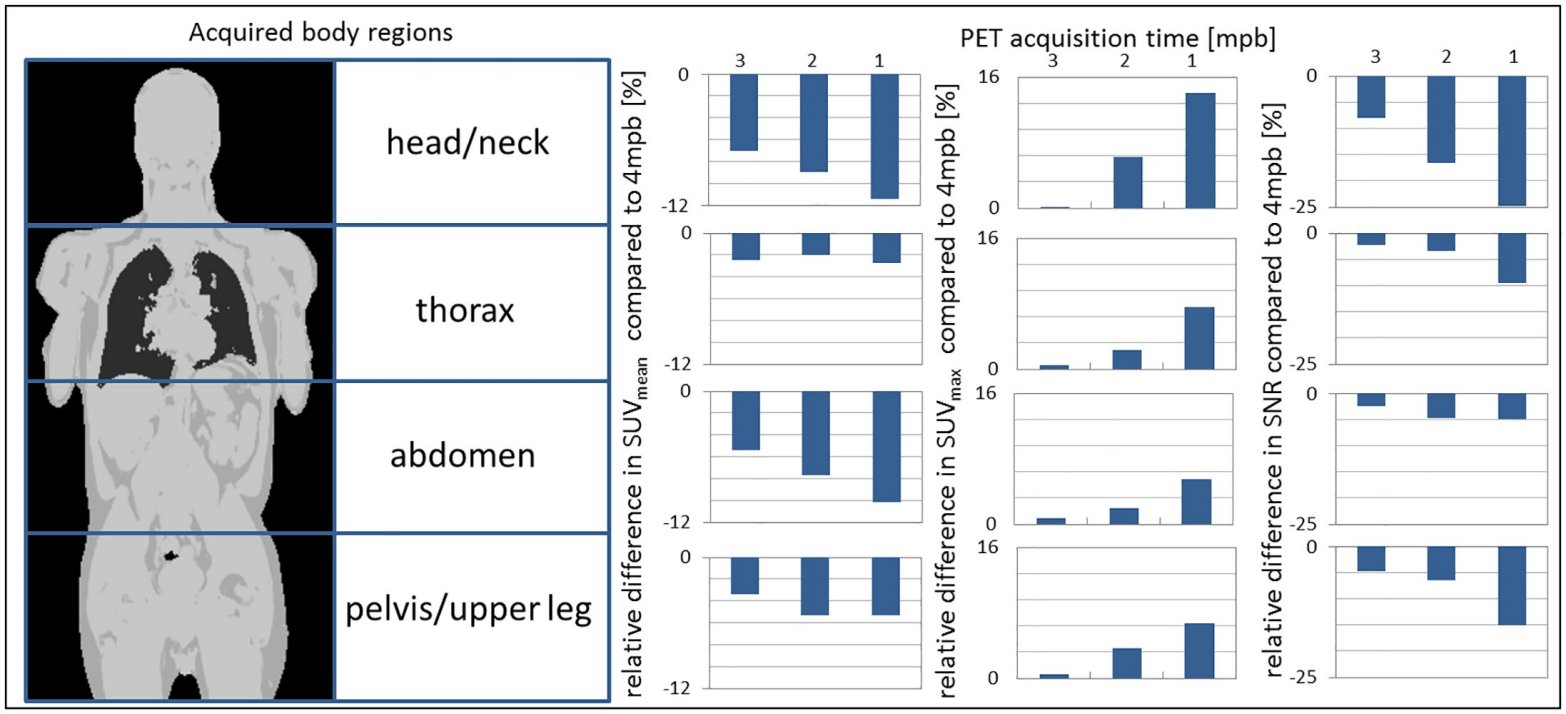
Image quality parameters in different body regions. Overview over the average standard-uptake-values (SUVs) SUV_mean_, SUV_max_ and the signal-to-noise ratio (SNR) for four different body regions (head/neck, thorax, abdomen, pelvis/upper legs). The relative difference in % is given for 3, 2 and 1 min per bed (mpb) PET acquisition time in comparison to 4 mpb serving as the reference standard. The impact of reduced PET acquisition times on image quality and quantification parameters is lower for the thorax and pelvic body regions.

The detected lesions were sorted into four different body regions: head/neck, thorax, abdomen and pelvis/upper legs. In Fig 2 an overview over SUV_mean_, SUV_max_ and the SNR for these different body regions is shown. The relative difference in % is given for 3, 2 and 1 min per bed PET acquisition time in comparison to 4 mpb serving as the reference standard. In the head/neck region the differences in SUVs and SNR between 4 mpb and shorter reconstructed acquisition times is highest. In the thoracic region those differences in SUV_mean_ are minimal, whereas in the abdominal region SUV_max_ and SNR are minimal.

Due to the heterogeneous patient cohort a large variance of some important biological image quality determinants are given. Besides the PET acquisition time also injected activity, patients’ BMI and the time between injection and examination might have an impact on PET image quality. The image quality analysis for different subgroups is shown in Table 4. Subgroups are built to show the impact of different BMIs, post injection times and applied activity of image quality parameters. The patient data was rearranged in these three categories: BMI, post injection time and applied radiotracer dosage, by comparing each value to the ‘mean value’ and by arranging the data in ‘category > Ø’ or ‘category < Ø’. The ‘mean value’ given in the table represents the average over all patients. In each subgroup the image quality parameters were analyzed. A lower BMI, short time interval between activity injection and PET/MR examination and a higher activity are optimal conditions to obtain high image quality regardless of different PET reconstruction times. For example, the image noise decreases with BMI < 25.5 kg/m^2^ from 42.1% (mean value over all patients) to 39.6% in the 1 mpb reconstruction, while it increases in patients with BMI > 25.5 kg/m^2^ to 43.1%. Shorter post injection times also result in higher image quality e.g. with regard to SNR, which increases in primary PET/MR examinations from 9.3 to 10.6 in the 1 mpb reconstruction. The effect is also visible in applied activity. The CNR decreases from 20.5 to 20.0 in 1 mpb reconstruction with lower activity.

**Table 4:** Image quality parameters for different PET acquisition times arranged for subgroups.

	1 min			2 min			3 min			4 min		
	noise (blood pool) [%]	SNR	CNR	noise (blood pool) [%]	SNR	CNR	noise (blood pool) [%]	SNR	CNR	noise (blood pool) [%]	SNR	CNR
BMI < Ø	39,6 ± 12,9	9,7 ± 1,6	20,8 ± 6,9	31,8 ± 10,1	11,3 ± 1,4	22,0 ± 6,5	24,5 ± 13,1	13,3 ± 1,9	23,8 ± 10,3	20,2 ± 11,1	13,9 ± 2,9	27,4 ± 8,1
primary PET/MR	40,0 ± 14,1	10,6 ± 1,9	21,5 ± 6,1	31,5 ± 10,3	10,9 ± 2,0	21,4 ± 8,0	25,6 ± 12,8	12,9 ± 2,1	23,1 ± 9,8	21,0 ± 10,7	13,3 ± 2,7	27,4 ± 7,7
activity > Ø	39,2 ± 12,2	9,9 ± 1,9	20,6 ± 9.0	30,6 ± 11,6	11,1 ± 1,9	20,8 ± 8,2	23,8 ± 11,9	14,0 ± 2,4	23,3 ± 10,7	19,7 ± 11,0	13,6 ± 3,0	26,9 ± 8,0
mean value	42,1 ± 14,9	9,3 ± 1,9	20,5 ± 9,9	33,6 ± 10,6	10,4 ± 1,6	20,8 ± 9,2	26,0 ± 12,2	12,9 ± 2,1	22,9 ± 11,7	22,2 ± 10,3	13,1 ± 2,7	26,6 ± 8,1
BMI > Ø	43,1 ± 11,3	7,8 ± 1,2	18,9 ± 11,7	33,9 ± 12,7	9,2 ± 1,8	19,1 ± 10,4	29,1 ± 13,6	12,7 ± 2,0	20,8 ± 11,4	24,9 ± 12,9	11,8 ± 2,5	26,0 ± 10,1
non primary PET/MR	43,9 ± 13,9	9,1 ± 1,5	19,0 ± 10,6	33,2 ± 10,3	10,0 ± 1,4	19,0 ± 11,1	26,9 ± 14,4	12,8 ± 1,7	21,1 ± 11,4	22,9 ± 12,2	12,0 ± 2,9	25,9 ± 9,3
activity < Ø	48,0 ± 13,1	8,9 ± 0.9	20,0 ± 11,2	34,6 ± 10,6	9,9 ± 1,4	19,7 ± 10,1	28,4 ± 12,3	11,7 ± 1,8	22,5 ± 12,2	25,1 ± 11,8	12,6 ± 2,1	26,4 ± 9,6

Image quality parameters (mean ± SD) for different PET reconstruction times are shown. The ‘mean value’ represents the average over all patients. Subgroups are built to show the impact of different body-mass-indexes (BMI), post injection times and applied activity of the image quality parameters image noise, signal-to-noise ratio (SNR) and contrast-to-noise ratio (CNR).

The correlation coefficients between the image quality determinants PET acquisition time, applied dose, post injection time and BMI on PET image quality parameters SNR, CNR and image noise (blood pool) are given in Table 5. Acquisition time and dose both exhibit a positive correlation on SNR and CNR (p < 0.01) and a negative correlation on the image noise (p < 0.01). Post injection time showed no significant correlation between any of the image quality parameters. The BMI reveals a significant negative correlation with SNR and a significant positive correlation with the image noise (p < 0.05).

**Table 5:** Correlation coefficients between image quality determinants and PET image quality parameters.

	acquisition time	dose	post injection time	BMI
SNR	0,746	0,756	-0,173	-0,584
CNR	0,616	0,560	-0,236	-0,319
image noise	-0,741	-0,648	0,311	0,661

The correlation coefficients between the image quality determinants PET acquisition time, applied dose, post injection time and body-mass-index (BMI) on PET image quality parameters signal-to-noise ratio (SNR), contrast-to-noise ratio (CNR) and image noise (blood pool) are given.

**Fig 3:**
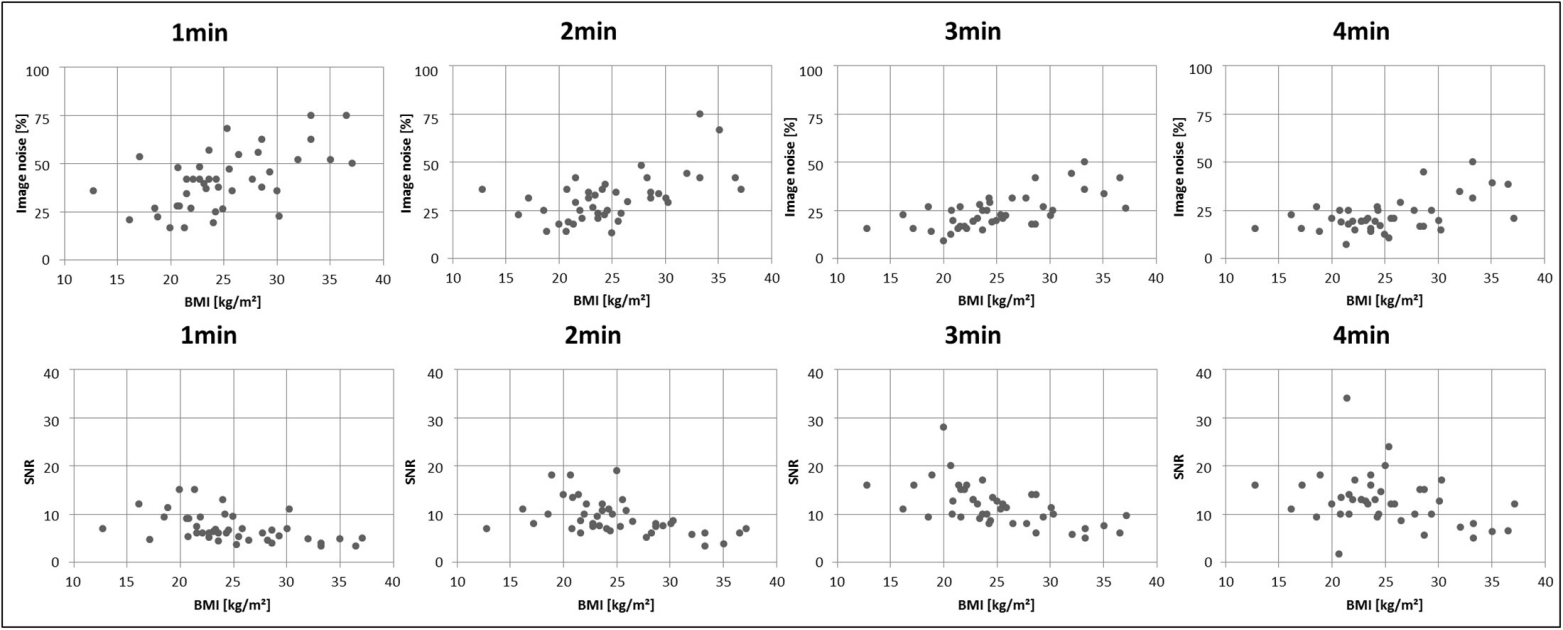
Correlation of two image quality parameters against BMI for different PET acquisition times. Correlation graphs of two image quality parameters. The image noise in the blood pool and the signal-to-noise ratio (SNR) against the body-mass-index (BMI) of each patient is compared for 1, 2, 3 and 4 minutes per bed position PET acquisition. Note that image noise decreases with longer PET acquisition times, while SNR increases.

Fig 3 depicts the correlation of the image quality parameters image noise (liver) and SNR against BMI of each patient for different reconstruction times. The image noise increases, while SNR decreases for shorter PET acquisition times. The image noise is higher in patients with larger BMI, while SNR is lower in those patients.

**Fig 4:**
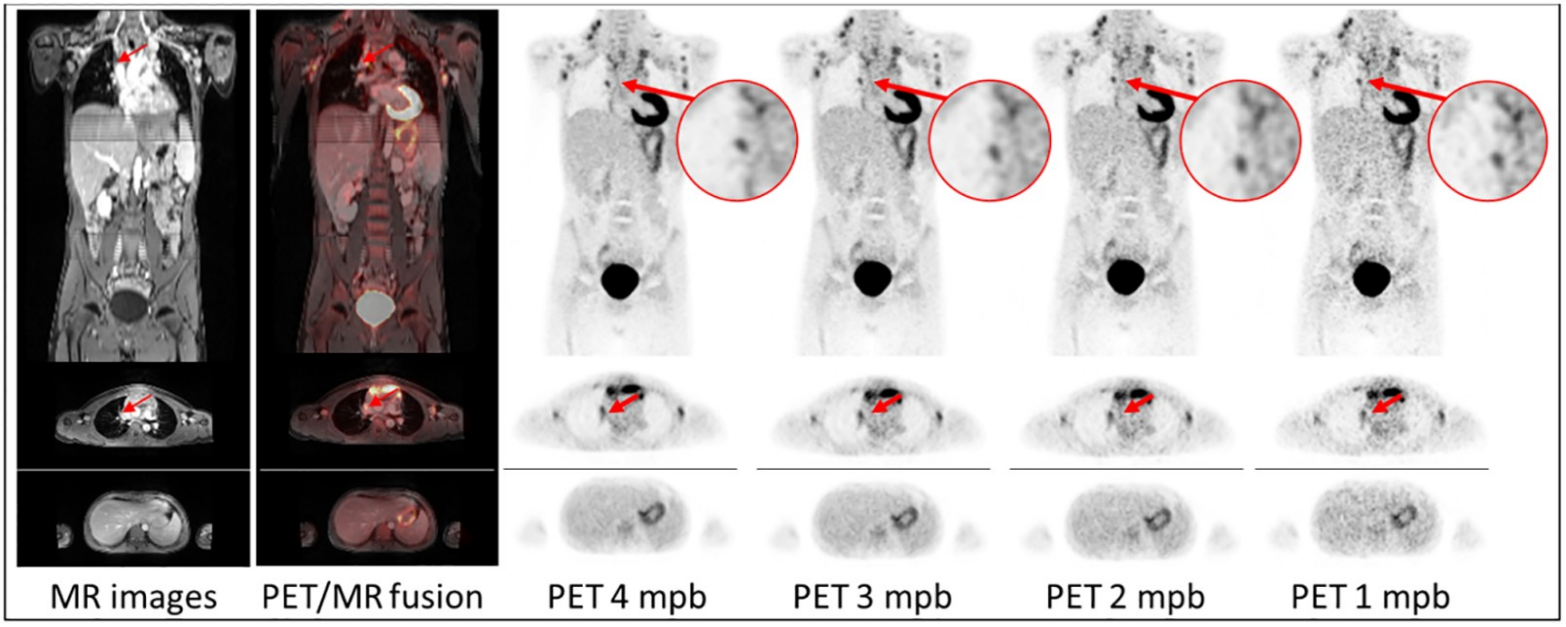
Patient example of consistently good image quality for different PET acquisition times. Example of consistently good image quality. MR images, PET/MR fusion images and PET images of patient #10 after 4, 3, 2 and 1 minutes per bed (mpb) position in coronal and axial orientation. The lesion is marked (red arrow). In addition an axial slice of the liver is shown. Discrete stripe artifacts in MR images result from coronal reformats at the positions where the transaxially acquired slice stack of two neighbored bed positions in the thorax are merged. Image noise (blood pool) increases from 18.7% at the 4 mpb to 23.7% in 1 mpb reconstruction. The CNR decreases from 28.3 at 4 mpb to 19.9 at 1 mpb. The SNR decreases from 14.8 at 4 mpb to 10.0 at 1 mpb.

Fig 4 depicts a patient example of consistently high image quality scores even for shorter PET acquisition times. The image noise (blood pool) slightly increases from 18.7% at the 4 mpb, 20.0% at 3 mpb, 22.3% at 2 mpb and to 23.7% in 1 mpb reconstruction. The CNR slightly decreases from 28.3 at 4 mpb, 23.1 at 3 mpb and 22.0 at 2 mpb, to 19.9 at 1 mpb. The SNR slightly decreases from 14.8 at 4 mpb, 13.7.0 at 3 mpb, 10.3 at 2 mpb, to 10.0 at 1 mpb. No remarkable changes in SUV_mean_ and SUV_max_ in different PET acquisition times are observable, indicating a low effect on PET quantification. The marked lesion with the lowest SUV_max_ of 9 is detectable in all PET reconstructions.

**Fig 5:**
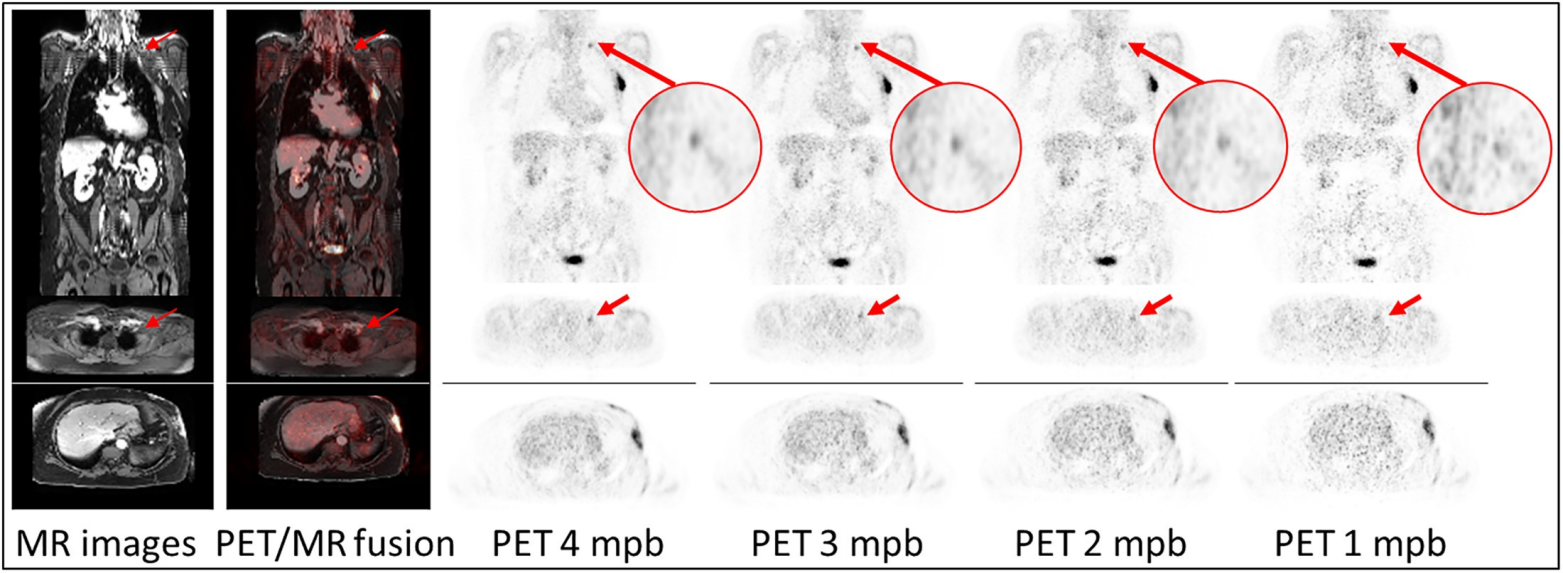
Patient example of decreasing image quality with shorter PET acquisition times. Example of decreasing image quality. MR images, fusion images and PET images of patient #24 after 4, 3, 2 and 1 minutes per bed (mpb) position in coronal and axial orientation. In addition an axial slice of the liver is shown. The lesion is marked (red arrow). Note that the marked lymph node lesion is not well detectable in PET images with 1 min timeframe. Discrete stripe artifacts in MR images result from coronal reformats at the positions where the transaxially acquired slice stack of two neighbored bed positions in the thorax are merged. Image noise (blood pool) increases from 28.1% at the 4 mpb to 58.1% in 1 mpb reconstruction. The CNR decreases from 21.1 at 4 mpb to 15.4 at 2 mpb. The SNR decreases from 6.6 at 4 mpb to 3.6 at 2 mpb.

Fig 5 shows a patient example of significantly decreasing image quality with shorter PET acquisition times. The image noise (blood pool) considerably increases from 28.1% at the 4 mpb, 38.9% at 3 mpb and 42.3% at 2 mpb, to 58.1% in 1 mpb reconstruction. The CNR significantly decreases from 21.1 at 4 mpb over 19.7 at 3 mpb to 15.4 at 2 mpb. The SNR decreases from 6.6 at 4 mpb over 5.3 at 3 mpb to 3.6 at 2 mpb. The marked lesion with a SUV_max_ of 9 in 4 mpb reconstruction is not detectable in the 1 min timeframe.

## Discussion

Aiming towards faster PET/MR imaging protocols, in this study the effect of reduced PET acquisition times on PET image quality and quantification parameters in a clinical whole-body PET/MR setting has been systematically investigated. List-mode format PET data of 51 patients with oncologic findings who underwent 18F-FDG whole-body PET/MR exams was reconstructed into different time frames to simulate reduced PET acquisition times. The PET data were then analyzed regarding image quality and quantification parameters.

As expected, PET image quality decreases with shorter PET image acquisition times. The subjective image quality score decreased from the 4 mpb to 1 mpb time frames. However, the average image quality of 2 mpb PET image time frames was rated as moderate, reflecting the second best out of four scores. Only the average IQS from the 1-minute timeframe was defined as poor. Overall, none of the reconstructed acquisition time frames was classified as non-diagnostic. The image noise in the blood pool, which is a quantitative parameter of image quality, increases towards shorter acquisition times from 22% at 4 mpb to 42% at 1 mpb. SNR and CNR slightly decrease with shorter acquisition time, but remain consistently high across all acquisition times.

The t-test has shown that there are no significant changes in image quality with regard to image noise, SNR and CNR between 3 and 4 minutes per bed position PET acquisition time. Whereas, toward shorter PET acquisition times the image quality decreases significant. The standard PET acquisition time in the actual whole-body PET/MR protocol is 4 minutes per bed position. Recent whole-body ultra-fast MR protocols in our clinical routine are shortened up to 3 minutes per bed MR acquisition. Calculating with 3 minutes per bed as the shortest time for MR acquisition in current PET/MR protocols, 1 minute per bed PET acquisition time could be saved to standard PET acquisition time (i.e. 25% of the total imaging protocol’s time is saved) without significant decrease in image quality. Further reduction of PET acquisition time is possible (up to 2 min per bed), but accompanied with significant losses in image quality.

The image quality metrics above strongly correlate with the lesion detectability in PET images. Despite the decreasing image quality with shorter PET acquisition time, no significant changes in SUV_mean_ and SUV_max_ were observable. The image quality was sufficient to detect all congruent lesions in all 4, 3, and 2-minute reconstructions by consensus reading. Only 2 lesions could not be detected (false-negative) when reading the 1 minute PET reconstruction intervals due to poor image quality. In general, the 2-minute acquisition time permitted correct detection of all lesions and, therefore, provides identical diagnostic information as the longer acquisition times of 3 and 4 minutes in this study.

The interpretation of variance factors of some important biological image quality determinants yields that PET image quality is mainly influenced by two key factors: the PET acquisition time and the injected tracer activity. The simplified factor analysis shows the highly significant (p < 0.01) correlation between PET acquisition time and applied activity on PET image quality. Both, acquisition time and dose exhibit a positive correlation on SNR and CNR and a negative correlation on image noise. Whereas, the post injection time showed no significant correlation between any of the image quality parameters. In the context of our study, it seems to have no impact on PET image quality whether the patients had a consecutive PET/CT examination, subsequently referred to PET/MR, or a PET/MR only examination. Another determinant exhibiting a significant correlation with PET image quality is the patients’ BMI. The BMI reveals a significant negative correlation with SNR and a significant positive correlation with the image noise (p < 0.05). A higher BMI seems to reduce PET count statistics, and therefore PET image quality. Accordingly, for patients with a high BMI it could be beneficial to perform a 4 minutes standard PET acquisition.

Figs 4 and 5 provide example cases for best and worst rated image quality (Figs 4 and 5). Patient #10 in Fig 4 is a 14-year-old child with a rather low BMI of 16.2 kg/m^2^. The PET/MR measurement here started comparably early at 63 minutes post injection. In this example, CNR and SNR were measured higher than the average values determined in this study, whereby the image noise was lower than the average over all patients. The same trend of increased SNR and CNR and decreased image noise is observable in five further patients under 15 years, who all have a BMI lower than 20 kg/m^2^ and a post injection time below 80 minutes. At the other end of the patient spectrum, Fig 5 provides an example of patient 5, who is 74 years old with a rather high BMI of 32.1 kg/m^2^. The PET/MR measurement here started comparably late at 148 minutes post injection. In this example, CNR and SNR were measured lower than the average values in this study, whereby the image noise was higher than the average over all patients. From these observations, we conclude that a low BMI and a short time interval between activity injection and PET/MR examination are optimal conditions for obtaining high image quality, SNR and CNR. This is to be expected when considering factors like tracer half-life time and count-rate attenuation in tissue. This conclusion can be derived from the data in Table 4 and Fig 3. Besides the two key factors acquisition time and injected activity, PET image quality is influenced by the BMI. A high BMI, a long post injection time and less injected activity worsen the resulting PET image quality with regard to higher image noise and lower SNR and CNR values.

Our study includes 51 patients with a broad range of different oncologic findings. The study setup with retrospective reconstruction of PET data in list-mode format into different time-intervals allowed for a systematic and controlled evaluation of image quality and quantitative parameters while comparing to the fully sampled 4-minute PET data set as standard of reference. This allows eliminating numerous confounding factors that would have been present when multiple tracer injections and PET re-examinations would have been performed. Additionally, the study setup eliminated the influence of tracer wash-in and wash-out that is inherently present in all comparison studies that employ re-examinations with resulting different time points after tracer injection.

The broad range of clinical indications in this retrospective PET/MR study might be considered as a limitation. Due to the heterogeneous patient cohort a large variance of some important biological image quality determinants are given. Besides the PET acquisition time also the injected activity, patients’ BMI and the time between injection and examination etc. might have an impact on PET image quality. Thus, the power of statistical analyses in patient data set with such variance may be inherently low. In this patient cohort the decrease of PET image quality due to shorter PET acquisition time (2 min or less) was significant. Also the correlation of PET acquisition time, dose and BMI on PET image quality was proven to be significant in this patient cohort.

The simple gating of PET list-mode data, where only the first part of the 4-minute measurements was reconstructed into shorter 3, 2, 1-minute time intervals could be considered a constraint of this study. Instead of progressively shortening the acquisition time intervals from 4-minute to 1-minute intervals as in this study, the method of retrospective randomized undersampling of list-mode would additionally account for potential patient motion or in cases where high tracer dynamics are to be expected [22] [23].

Several recent studies have investigated the possibility to reduce the number of potentially redundant MR sequences with the overall aim to shorten the overall PET/MR acquisition time while maintaining diagnostic PET/MR information [11]–[17]. Nevertheless, the PET/MR acquisition time in these fast protocols was still 4 minutes per bed position, so that simultaneous MRI can still be considered as the time limiting factor. The ultimate short whole-body PET/MR protocol is the dual use of the Dixon AC sequence also as anatomic MR imaging correlate without adding further diagnostic MR sequences. Such a fast protocol has been investigated by Eiber et al. [17]. The conclusion was, that the fast protocol using MR-based AC only provides comparable results as a low dose PET/CT [17]. Such a protocol, theoretically, could be as fast as 19 seconds per bed, the duration for a low spatial resolution MR Dixon A protocol [3] [1]. In such a scenario, PET might be the time limiting factor in PET/MR. In the present study we have investigated that 2 minutes PET acquisition per bed position is sufficient to provide accurate lesion detection and adequate image quality. Further reduction of PET acquisition time in a comparable setting may result in the loss of diagnostic information due to poor PET image quality.

Another indication of this study is that radiotracer dose may be further reduced in 18F-FDG whole-body PET/MR in patients with oncologic findings while maintaining high image quality and accurate PET quantification. Generally, PET image quality is influenced by mainly two key factors: the PET acquisition time and the injected tracer activity. The comparatively long PET/MR examination times of current protocols may be transformed into an advantage since longer PET acquisition times provide better count statistics and image quality. Increasing the PET acquisition time to match the prolonged MR examination times may allow a decrease of the applied radiotracer activity. Oehmigen et al. [24] have shown in systematic NEMA phantom experiments under controlled conditions that reducing the PET acquisition time by half provides equivalent PET image quality as doubling acquisition time with half the injected radiotracer dose [24]. According to the results of the present study, a reduction of the radiotracer dose by a factor of two seems practical when extending the PET acquisition time from 2 min to 4 min while maintaining all other PET imaging parameters and confounding factors.

## Conclusion

Reconstruction of whole-body PET data with different time intervals has shown that 2 minutes instead of 4 minutes acquisition time per bed position is sufficient to provide accurate lesion detection, high image quality, SNR and CNR in a clinical PET/MR setting despite the expected trends to lower image quality with shorter PET acquisition times. These findings provide the foundation for the potential to reduce the PET acquisition time in fast 18F-FDG whole-body PET/MR imaging protocols. Beside the two key factors PET acquisition time and applied activity, PET image quality is also influenced by the patients’ BMI.

## Supporting information

S1 Table Measured standardized uptake values in all patient data sets. (XLSX)
